# Structural study and thermodynamic characterization of inhibitor binding to lumazine synthase from *Bacillus anthracis*

**DOI:** 10.1107/S0907444910029690

**Published:** 2010-08-13

**Authors:** Ekaterina Morgunova, Boris Illarionov, Sabine Saller, Aleksander Popov, Thota Sambaiah, Adelbert Bacher, Mark Cushman, Markus Fischer, Rudolf Ladenstein

**Affiliations:** a Karolinska Institutet NOVUM, Center of Structural Biochemistry, Hälsovägen 7–9, 141 57 Huddinge, Sweden; b Institut für Lebensmittelchemie, Universität Hamburg, Grindelallee 117, 20146 Hamburg, Germany; c European Synchrotron Radiation Facility, BP 220, F-38043 Grenoble CEDEX 09, France; d Department of Medicinal Chemistry and Molecular Pharmacology, Purdue University, USA; e Chemistry Department, Technical University of Munich, 85747 Garching, Germany

**Keywords:** *Bacillus anthracis*, riboflavin biosynthesis, lumazine synthase, anthrax, inhibition, drug design

## Abstract

The crystal structure of lumazine synthase from *Bacillus anthracis* was solved by molecular replacement and refined to *R*_cryst_ = 23.7% (*R*_free_ = 28.4%) at a resolution of 3.5 Å. The structure reveals the icosahedral symmetry of the enzyme and specific features of the active site that are unique in comparison with previously determined orthologues. The application of isothermal titration calorimetry in combination with enzyme kinetics showed that three designed pyrimidine derivatives bind to lumazine synthase with micromolar dissociation constants and competitively inhibit the catalytic reaction. Structure-based modelling suggested the binding modes of the inhibitors in the active site and allowed an estimation of the possible contacts formed upon binding. The results provide a structural framework for the design of antibiotics active against *B. anthracis*.

## Abstract 2

Crystallographic studies of lumazine synthase, the penultimate enzyme of the riboflavin-biosynthetic pathway in *B. anthracis*, provide a structural framework for the design of antibiotic inhibitors, together with calorimetric and kinetic investigations of inhibitor binding.

## 1. Introduction

**Figure 1:**
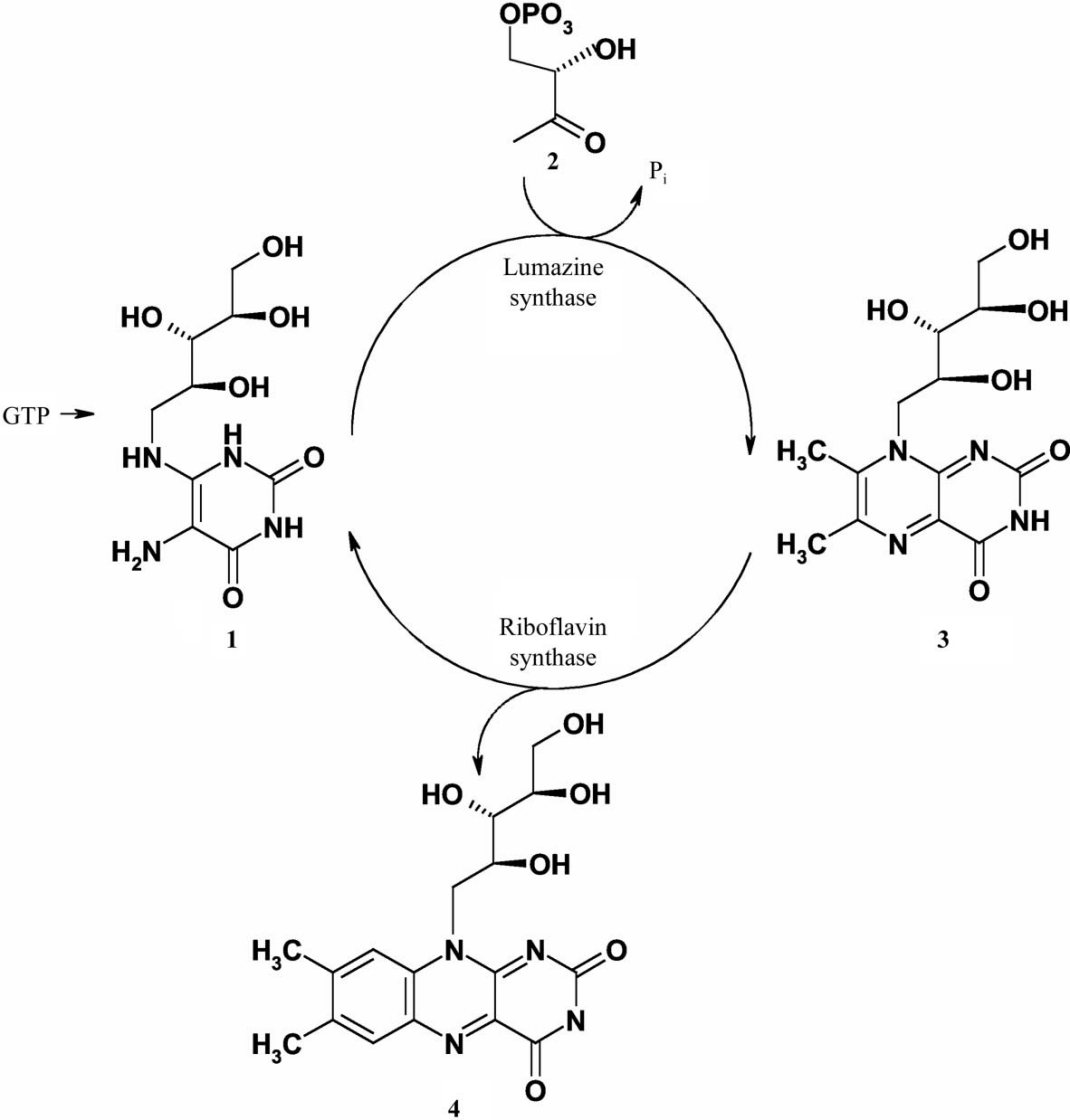
Terminal steps of riboflavin biosynthesis. **1**, 5-Amino-6-ribitylamino-2,4(1*H*,3*H*)-pyrimidinedione; **2**, 3,4-dihydroxy-2-butanone-4-phosphate; **3**, 6,7-dimethyl-8-ribityl-lumazine; **4**, riboflavin.

Riboflavin (compound **4**, Fig. 1 ▶) is essential in all organisms as a precursor for flavocoenzymes. With the exception of *Listeria monocytogenes*, pathogenic bacteria biosynthesize riboflavin *de novo*, whereas animals depend on nutritional sources. In certain bacterial pathogens (*Salmonella*, *Brucella*), enzymes of the riboflavin-biosynthesis pathway have documented virulence-factor status. Owing to the absence of the riboflavin-biosynthesis pathway in animals, the enzymes of the pathway are potential targets for anti-infective therapy and riboflavin-biosynthesis inhibitors should be exempt from target-related toxicity.

The biosynthesis of riboflavin has been studied in considerable detail and it is known that the early steps show significant differences between taxonomic kingdoms, although the final two steps, namely the formation of 6,7-dimethyl-8-ribityl­lumazine (compound **3**, Fig. 1 ▶) and riboflavin (compound **4**), are both strictly conserved in all organisms. Six enzymes are known to date to be involved in the riboflavin-biosynthetic pathway in eubacteria: GTP cyclohydrolase II, 2,5-diamino-6-ribosylamino-4(3*H*)-pyrimidinone 5′-phosphate deaminase, 5-­amino-6-ribosylamino-2,4(1*H*,3*H*)-pyrimidinedione 5′-phosphate reductase, 3,4-dihydroxy-2-butanone 4-phosphate syn­thase, 6,7-dimethyl-8-ribityllumazine synthase (LS) and riboflavin synthase. LS and riboflavin synthase catalyze the terminal reactions in the process. More specifically, LS catalyzes the condensation of 5-amino-6-ribityl­amino-2,4(1*H*,3*H*)-pyrimidinedione (compound **1**, Fig. 1 ▶) with 3,4-dihydroxy­butanone 4-phosphate (compound **2**), affording 6,7-dimethyl-8-ribityllumazine (compound **3**), and riboflavin synthase catalyzes the dismutation of two molecules of compound **3** to form one molecule each of riboflavin (compound **4**) and compound **1**.

**Figure 2:**
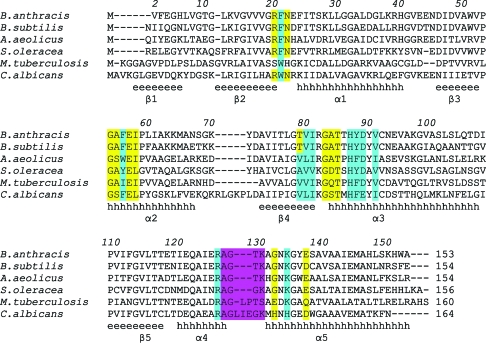
Amino-acid sequence alignment of the icosahedral lumazine synthases from *B. anthracis*, *B. subtilis*, *A. aeolicus* and *S. oleracea* with two pentameric lumazine synthases from *M. tuberculosis* and *C. albicans*. The numbering corresponds to the enzyme from *B. anthracis*. The secondary-structure elements are shown below the sequences as they are found in BaLS. The residues highlighted in yellow and cyan are involved in the formation of the active site and the binding of inhibitors, respectively. The part of the structure which is responsible for the formation of the icosahedral assembly is highlighted in magenta.

Structural, biochemical and genetic studies of LS have a long and successful history. Structures of LS from different organisms have been solved at near-atomic resolution (Ritsert *et al.*, 1995 ▶; Persson *et al.*, 1999 ▶; Meining *et al.*, 2000 ▶; Braden *et al.*, 2000 ▶; Liao *et al.*, 2001 ▶; Zhang *et al.*, 2001 ▶; Gerhardt *et al.*, 2002 ▶; Morgunova *et al.*, 2005 ▶, 2007 ▶). The enzymes show 30–48% sequence identity (Fig. 2 ▶) and occur in three oligomerization states. LS from *Saccharomyces cerevisiae*, *Schizosaccharomyces pombe*, *Magnaporthe grisea*, *Mycobacterium tuberculosis* and *Candida albicans* assembles into homopentamers (Persson *et al.*, 1999 ▶; Meining *et al.*, 2000 ▶; Gerhardt *et al.*, 2002 ▶; Morgunova *et al.*, 2005 ▶, 2007 ▶). LS from *Brucella abortus* has been shown to form *D*_5_-symmetric dimers of pentamers (Klinke *et al.*, 2005 ▶). The enzymes from *Bacillus subtilis*, *Aquifex aeolicus*, *Escherichia coli* and *Spinacia oleracea* form icosahedral capsids constituted of 60 identical subunits, which can be described as dodecamers of pentamers. Moreover, *B. subtilis* also forms a 1 MDa enzyme complex consisting of an LS capsid with three riboflavin synthase subunits enclosed in the central core (Ladenstein *et al.*, 1988 ▶; Ritsert *et al.*, 1995 ▶). The topology of the homopentameric enzymes resembles that of the pentameric building blocks of the icosahedral and decameric enzymes.

Catalysis occurs in equivalent active sites, which are located at the interfaces of adjacent subunits in the pentamers. Based on the structures of the substrates and product of the reaction catalyzed by LS, numerous organic compounds have been synthesized and characterized kinetically as well as thermodynamically as inhibitors of LS from *M. tuberculosis*, *Brucella abortus* and *C. albicans* (Cushman *et al.*, 1997 ▶, 2001 ▶, 2002 ▶, 2004 ▶, 2005 ▶; Cushman, Mavandadi *et al.*, 1999 ▶; Cushman, Mihalic *et al.*, 1999*a* ▶,*b* ▶; Braden *et al.*, 2000 ▶; Chen *et al.*, 2005 ▶; Zhang *et al.*, 2008 ▶).

Whereas several biosynthetic pathways of *Bacillus anthracis* have been investigated in some detail, very little is known about riboflavin biosynthesis in this bacterium. In order to suggest possible lead compounds for potential drugs against this pathogen, we determined the three-dimensional structure of *B. anthracis* lumazine synthase (BaLS) and performed kinetic assays, isothermal titration calorimetry binding studies and structure-based modelling for several synthetic ligands.

## 2. Material and methods

### 2.1. Cloning and bacterial cell culture

In order to construct an open reading frame for the expression of BaLS, we cloned the orthologous gene of *B. cereus* while replacing the codon for the single amino-acid residue that differs between the two orthologues. Specifically, we amplified the *B. cereus* gene using the oligonucleotides BARibH-Rbs-*Eco*RI and BARibH-*Bam*HI-Hi (designed to introduce the desired Q151H replacement) as PCR primers (see Table 1 ▶). The amplificate was purified and digested with *Eco*RI and* Bam*HI. The fragment was cloned into the pNCO-113 plasmid which had been treated with the same enzymes. The recombinant plasmid carrying the BaLS gene was transformed into XL1 *E. coli* cells. The plasmid was re-isolated and transformed into *E. coli* M15 [pREP4] cells (Stüber *et al.*, 1990 ▶) carrying the pREP4 repressor plasmid for the overexpression of *lac* repressor protein, where it directed the synthesis of full-length BaLS (without tags or any other additions). Kanamycin (15 mg l^−1^) and ampicillin (170 mg l^−1^) were added to secure the retention of both plasmids in the host strain. The cultures were incubated at 310 K with shaking. At an optical density of 0.7 (at 600 nm), isopropyl β-d-1-thiogalactopyranoside was added to a final concentration of 2 m*M* and the cultures were incubated for 5 h at 310 K with shaking. The cells were harvested by centrifugation, washed with 0.9%(*w*/*v*) sodium chloride and stored at 253 K.

### 2.2. Purification

All purification steps were performed at 277 K. Frozen cell mass (∼5 g) was thawed in 30 ml 50 m*M* potassium phosphate pH 8.0 containing 10 m*M* EDTA. The suspension was ultrasonically treated and centrifuged. The supernatant was passed through a column of Q Sepharose (5 × 10 cm; Amersham Pharmacia Biotech, Freiburg, Germany) which had been equilibrated with 20 m*M* potassium phosphate pH 8.0 (buffer *A*). The column was washed with 100 ml buffer *A* and developed with a linear gradient of 20–1000 m*M* potassium phosphate pH 8.0 in a total volume of 900 ml. The fractions were combined, concentrated by ultrafiltration and dialyzed against 100 m*M* potassium phosphate pH 8.0 (buffer *B*). The solution was passed through a column of Superdex 200 (2.6 × 60 cm; Amersham Pharmacia Biotech, Freiburg, Germany) which had been equilibrated with buffer *B*. The protein was eluted with buffer *B* and concentrated by ultrafiltration.

### 2.3. Protein sequencing

Sequence determination was performed by the automated Edman method using a 471A Protein Sequencer (Perkin–Elmer).

### 2.4. Inhibitors

4-(6-Chloro-2,4-dioxo-1,2,3,4-tetrahydropyrimidine-5-yl)-*n*-butyl 1-phosphate (JC33), 5-(6-chloro-2,4-dioxo-1,2,3,4-tetrahydropyrimidine-5-yl)-*n*-pentyl 1-phosphonate (JC72) and 5-(6-chloro-2,4-dioxo-1,2,3,4-tetrahydropyrimidine-5-yl)-6-keto-*n*-hexyl 1-phosphate (TS23) were prepared as described elsewhere (Cushman, Mihalic *et al.*, 1999*b* ▶; Cushman *et al.*, 2004 ▶, 2005 ▶).

### 2.5. Crystallization and data collection

BaLS crystals were grown by the vapour-diffusion technique in sitting drops by the following procedure: 1 µl protein solution (17 mg ml^−1^) in 100 m*M* potassium phosphate pH 8.0 was mixed with 1 µl reservoir solution (100 m*M* Tris–HCl pH 8.0, 36% polypropylene glycol P400 and 20 m*M* DTT). Thin fragile plate-shaped crystals appeared in one month and grew to dimensions of 0.05 × 0.1 × 0.4 mm in several weeks.

X-ray diffraction data were collected from a single crystal on beamline ID23-1 at the European Synchrotron Light Source (ESRF, Grenoble, France) at 100 K using the reservoir solution as a cryoprotectant. The data-collection strategy was optimized with the program *BEST* (Bourenkov & Popov, 2006 ▶). The data were integrated with the program *XDS* (Kabsch, 1988 ▶, 2010 ▶) and scaled with *SCALA* (Collaborative Computational Project, Number 4, 1994 ▶). The crystals belonged to the ortho­rhombic system, space group *P*2_1_2_1_2, with unit-cell parameters *a* = 157.2, *b* = 222.3, *c* = 473.5 Å. Statistics of data collection are presented in Table 2 ▶.

### 2.6. Structure determination

The structure of BaLS was solved by molecular replacement using the programs *MOLREP* and *FFFEAR* as implemented in *CCP*4 (Collaborative Computational Project, Number 4, 1994 ▶) with the structure of a half icosahedron of *B. subtilis* LS (PDB code 1rvv; Ritsert *et al.*, 1995 ▶) as a Patterson search model.

### 2.7. Refinement

The initial model consisting of three halves of the icosahedral particle, each of which contained six pentamers, was subjected to rigid-body refinement with *PHENIX* (Adams *et al.*, 2002 ▶) and *REFMAC* (Collaborative Computational Project, Number 4, 1994 ▶). A special version of *REFMAC* was used which could handle 150 000 non-H atoms. Solvent flattening and histogram matching were applied to the initial electron density with the program *DM* as implemented in *CCP*4 (Collaborative Computational Project, Number 4, 1994 ▶). Noncrystallographic averaging was performed between the 18 pentamers belonging to the three half icosahedra using the program *DM*. The mask covering one pentamer was calculated with *NCS-MASK* (Collaborative Computational Project, Number 4, 1994 ▶) and the noncrystallographic sym­metry operators were improved after every cycle of averaging. The procedure improved the initial electron-density map and allowed the building of almost all of the residues that had been replaced by alanine in the original model. The model was rebuilt with the graphics programs *O* (Jones *et al.*, 1991 ▶) and *Coot* (Emsley & Cowtan, 2004 ▶). Further refinement was performed with *REFMAC* and *phenix.refine* using TLS options and noncrystallographic restraints between pentamers inside the icosahedral particle and between subunits in one pentamer. The progress of refinement was monitored by the free *R* factor using 2% (4118 reflections) of the data put aside from the calculations. The difference |*F*_o_| − |*F*_c_| electron-density maps showed positive 5σ peaks in each active site near the positions of phosphate ions or phosphate-group binding sites in the other known LS structures. Since the protein was isolated and stored in 100 m*M* potassium phosphate buffer, we interpreted these peaks as phosphate ions. The final model consisting of 90 protein subunits and 90 phosphate ions was refined at a resolution of 3.5 Å to *R*_cryst_ = 23.7% and *R*_free_ = 28.4%. The atomic coordinates and structure factors of BaLS have been deposited in the Protein Data Bank (three files with accession codes 1vsx, 1vsw and 3jv8).

### 2.8. Isothermal titration calorimetry (ITC)

Calorimetric measurements were carried out on a VP Iso­thermal Titration Calorimeter (MicroCal Inc., Northampton, Massachusetts, USA) calibrated with standard electrical pulses. Solutions containing 0.03 m*M* BaLS (on the basis of monomers) and 50 m*M* potassium phosphate pH 7.0 were titrated with 5 m*M* inhibitor in the same buffer. All solutions were degassed by stirring under vacuum before use. Titrations were performed at 303 K with injected aliquots of 4 µl inhibitor solution. A total of 25–30 injections were made, with 240 s between injections. In control experiments the inhibitor was titrated against the buffer solution without protein in order to determine the dilution heat of the inhibitor. The background was subsequently subtracted from the test data involving BaLS. All data were evaluated using the *Origin* 5.0 software package (MicroCal). The apparent association constant *K*_a_ = 1/*K*_d_, binding enthalpy Δ*H* and stoichiometry *n*, together with their corresponding standard deviations, were determined by a nonlinear least-squares fit. The entropy and free energy of binding were obtained from the relation Δ*G* = −*RT*ln*K*_a_ = Δ*H* − *T*Δ*S*.

### 2.9. Kinetic assay of BaLS inhibitors

Assay mixtures contained 100 m*M* Tris–HCl pH 7.0, 100 m*M* NaCl, 5 m*M* dithiothreitol, 1%(*v*/*v*) DMSO, 100 µ*M* compound **2** (Fig. 1 ▶), 1 µ*M* BaLS (on the basis of monomers) and variable concentrations of compound **1** (3–80 µ*M*; Fig. 1 ▶) and inhibitor (0–45 µ*M*) in a volume of 0.2 ml. Assay mixtures were prepared as follows. A solution (170 µl) containing 105 m*M* Tris–HCl pH 7.0, 105 m*M* NaCl, 5.3 m*M* dithiothreitol, 118 µ*M* compound **2** and 1.18 µ*M* BaLS  (on the basis of monomers) was added to 10 µl DMSO containing 0–10 nmol inhibitor in a well of a 96-well microtitre plate. The reaction was started by adding 20 µl of a solution containing 105 m*M* Tris–HCl pH 7.0, 105 m*M* NaCl, 5.3 m*M* dithiothreitol and com­pound **1** (30–800 µ*M*). Formation of compound **3** was monitored photometrically at 408 nm and 300 K with a computer-controlled plate reader (∊_compound**3**_ = 10 200 *M* ^−1^ cm^−1^). Samples were measured at intervals of 20 s for a total period of 30 min.

### 2.10. Evaluation of kinetic data

The velocity/substrate-concentration data were fitted for all inhibitor concentrations with a nonlinear regression method using the program *DynaFit* (Kuzmic, 1996 ▶). Different inhibition models were considered for the calculation. *K*_i_ and *K*_is_ values ± standard deviations were obtained from the fit considering the most likely inhibition model.

### 2.11. Molecular modelling

**Figure 3:**
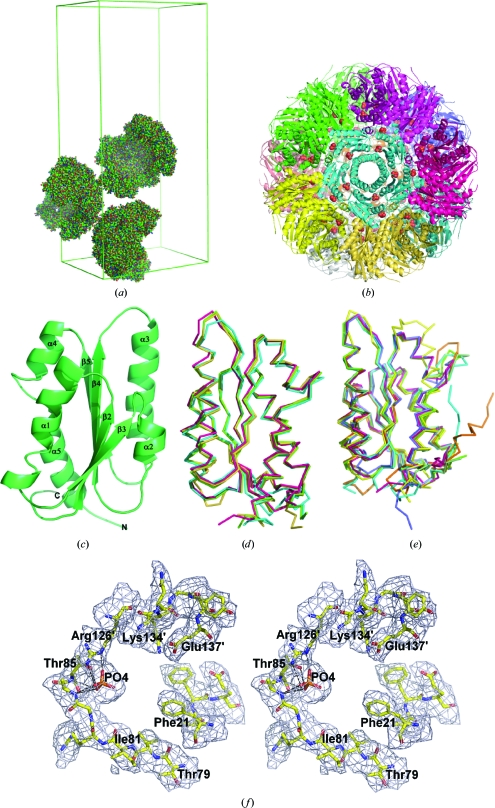
Structural diagrams of lumazine synthase from BaLS. (*a*) Packing of three icosahedral halves in the asymmetric unit of the *P*2_1_2_1_2 crystal lattice. (*b*) Icosahedral assembly of the enzyme. The view is along the fivefold icosahedral axis. Each pentamer is shown in a different colour and inorganic phosphate ions found in the active sites are shown as spheres. (*c*) Labelled secondary-structure arrangement of a monomer. (*d*) Structural comparison of BaLS with icosahedral LSs from *B. subtilis*, *A. aeolicus* and *Spinacia oleacea*; BaLS is shown in red. (*e*) Structural comparison of BaLS with pentameric enzymes from *M. tuberculosis*, *Schizosaccharomyces pombe*, *Saccharomyces cerevisiae* and *Brucella abortus*; BaLS is shown in red. (*f*) Stereodiagram of the 2|*F*_o_| − |*F*_c_| electron-density map around the active site of the enzyme with bound inorganic phosphate ion; the residues of a symmetry-equivalent adjacent subunit are labelled with primes.

The respective inhibitors were initially pre-docked into the active site of BaLS by the following procedure. The empty BaLS active site was structurally aligned with the structure of the active site of LS from *M. tuberculosis* (MtLS) in complex with JC33. Compounds JC72 and TS23 were first overlaid with JC33 in the active site of MtLS. The hypothetical complexes were structurally aligned with the empty BaLS dimer. The structural alignment was performed with the least-squares option (lsq) as implemented in *O*. The final docking of the inhibitor models to the binding site of BaLS was performed with *AutoDock* 3.0 (Morris *et al.*, 1998 ▶). The protein model was protonated and partial charges were added using *Chimera* (Pettersen *et al.*, 2004 ▶) and the solvation parameters were defined using the *AutoDock* 3.0 routine *Mol*2*topdbqs* (Wang *et al.*, 2006 ▶) after removal of the ions found in the structure. The respective inhibitor models were prepared for the docking procedure using *Chimera* (Pettersen *et al.*, 2004 ▶). The docking was performed on a rigid dimer of BaLS subunits that were directly adjacent in a pentamer module, while the ligand was allowed to adopt different conformations. The docking grid was centred on the centre of gravity of the putative binding site. All other parameters remained at their default values. 20 dockings for each inhibitor were calculated using the Lamarckian genetic algorithm (Morris *et al.*, 1998 ▶). In order to test the changes that occur in the conformations of protein side chains upon inhibitor binding, the most reliable models were subjected to 1000 steps of molecular-dynamics calculations followed by 300 steps of energy minimization. Molecular-dynamics calculations were performed with the program *CNS* (Brünger *et al.*, 1998 ▶) by using a simulated-annealing schedule in Cartesian coordinates at a constant temperature of 298 K, a dielectric constant of 1.0 for the protein and the nonbonded list cutoff of 13 Å as default values in *CNS*. The pictures in Figs. 3 and 7 were generated by *PyMOL* (DeLano, 2002 ▶).

## 3. Results and discussion

### 3.1. Protein purification

Full-length BaLS (without tags or other additions) was isolated from a recombinant *E. coli* strain as described in §[Sec sec2]2. The recombinant protein was purified by ion-exchange and gel-permeation chromatography and appeared to be homogeneous as judged by SDS–PAGE. The N-terminal sequence of the recombinant protein was verified by partial Edman degradation, affording the sequence motif MVFEGHLVGT, which was in perfect agreement with the translated open reading frame.

### 3.2. Overall protein structure and comparison with orthologous structures

BaLS was crystallized as described in §[Sec sec2]2. The three-dimensional structure was solved by molecular replacement using one half-molecule of the icosahedral *B. subtilis* LS (PDB code 1rvv; Ritsert *et al.*, 1995 ▶) as a Patterson search model. The asymmetric unit of the BaLS crystal contained 90 protein subunits of 16 255 Da molecular mass with a Matthews co­efficient of 2.83 Å^3^ Da^−1^ (*i.e.* three icosahedral half-molecules; see Fig. 3 ▶ *a*). Each BaLS molecule consists of 60 identical protein subunits arranged in 12 pentamers in accordance with icosahedral 532 symmetry (Figs. 3 ▶ *b* and 3 ▶ *c*). The BaLS monomer is comprised of 153 amino acids and shows the typical α/β/α-sandwich topology of known LS orthologues. The core of the protein subunit is formed by a four-stranded parallel β-­sheet which is flanked by α-helices (Fig. 3 ▶ *c*). Sequence (Fig. 2 ▶) and structural comparisons of the BaLS subunit with icosahedral orthologues showed a very high similarity for secondary-structure elements and only small differences in the conformations of the loops connecting β-­strands and α-helices (Fig. 3 ▶ *d*). On the other hand, comparison of the BaLS subunit with pentameric (non-icosahedral) LSs (Fig. 3 ▶ *e*) revealed more distinct differences in the loop regions, although the secondary-structure elements appeared to be rather conserved between icosahedral and pentameric enzymes.

The symmetry-related α_3_ helices of each pentameric ensemble of icosahedral BaLS surround a central channel. The central part of the channel wall is formed by the side chains of five Lys97 residues, creating a positively charged patch whose charge is compensated by the side chains of Glu94. The channel entrances are formed by the polar residues Asp89 and Asn93 and by Gln105 and Glu118, which face the solvent space and the particle core space, respectively. The amino-acid side chains inside the channel participate in stabilizing hydrogen-bond interactions. While the solvent content of the BaLS crystals could not be analyzed in detail, water molecules have been observed inside the homologous channels of other LS orthologues.

The N-terminus of each subunit forms an extra β-strand extending to the β-sheet of the adjacent subunit. 12 pentameric blocks make up one icosahedral particle with a diameter of about 157 Å, which is rather similar to the previously determined sizes of icosahedral LSs [160 Å for LS from *S. oleracea* (Persson *et al.*, 1999 ▶), 154 Å for LS from *A. aeolicus* (Zhang *et al.*, 2001 ▶) and 156 Å for LS from *B. subtilis* (Ritsert *et al.*, 1995 ▶)]. The characteristic icosahedral ionic contacts described in detail by Zhang *et al.* (2001 ▶) are well conserved in the BaLS structure, although there is one fewer positively charged Arg residue compared with LS from the hyperthermophilic bacterium *A. aeolicus*. The residues involved in the ionic interactions are Arg20, Arg39, Glu23, Asp35 and Glu144. The threefold interactions are formed by residues from helices α1 and α4. This contact is also well conserved in all known icosahedral LSs and is maintained by the hydrogen-bond network of three symmetry-equivalent Lys28 residues from neighbouring subunits on the surface of the capsid, by hydrophobic interactions involving Phe24, Ile120 and Ile124 and by three negatively charged Glu121 residues on the inner surface of the capsid. The twofold icosahedral axes at the interface between two pentamers are surrounded by residues from the end of strand β_4_ and the loop connecting helices α_4_ and α_5_. The interactions between pairs of adjacent subunits inside the pentamer are very extensive.

### 3.3. Active site

The cavities formed at the subunit interfaces are the active sites of lumazine synthase where both substrates (*i.e.* com­pounds **1** and **2**; Fig. 1 ▶) are bound. Notably, all icosahedral LSs including BaLS have 60 equivalent active sites. The heteroaromatic ring systems of compound **1** or substrate-analogous inhibitors are located in a hydrophobic pocket. Their ribityl side chain is embedded in a surface depression which is less accessible to solvent than the ring system and the alkylphos­phonyl or alkylphosphate chain. The binding of substrate-analogous inhibitors to LS appears to follow an induced-fit mechanism as follows. The phenyl ring of Phe20 swings into an orientation parallel to the heteroaromatic ring system of the inhibitors (π–π interaction), whereas in the native enzyme forms it is usually tilted away by more than 30° relative to this orientation. Position 20 is occupied by either a Phe or a Trp residue in orthologous LSs. This residue acts like a ‘gate’ controlling the path between the active-site cavity and the solvent environment. Arg125, which is involved in forming an ionic contact with the phosphonate or phosphate group of the inhibitors, is highly conserved throughout the compared orthologous LSs, like all other amino-acid side chains lining the wall of the active-site cavity. Arg125 forms a salt bridge with Glu131.

The structures of several complexes of LSs with substrate and product analogues (Ritsert *et al.*, 1995 ▶; Persson *et al.*, 1999 ▶; Meining *et al.*, 2000 ▶; Gerhardt *et al.*, 2002 ▶; Zhang *et al.*, 2003 ▶, 2008 ▶; Klinke *et al.*, 2005 ▶; Morgunova *et al.*, 2005 ▶, 2006 ▶, 2007 ▶) together with mutation analysis performed for *B. subtilis* LS showed that residues Phe21, Phe59, Thr79, Ile81, Thr85 and His87 from one subunit, and residues Arg126, Lys134 and Glu137 (BaLS numbering) from the adjacent subunit surprisingly have only a weak influence on substrate binding and catalysis (Kis & Bacher, 1995 ▶; Kis *et al.*, 1995 ▶), pointing to the control of activation entropy (Fischer *et al.*, 2003 ▶) and the proximity effect as the main driving forces in LS catalysis. In the absence of substrates or products a phosphate ion usually remains bound at the putative location of the substrate (compound **2**, Fig. 1 ▶; Persson *et al.*, 1999 ▶; Braden *et al.*, 2000 ▶; Zhang *et al.*, 2001 ▶; Morgunova *et al.*, 2007 ▶). BaLS was crystallized from phosphate buffer. In the resulting structure, we found electron-density peaks at the 5σ level in each of the 90 active sites in the asymmetric unit. These densities were interpreted and refined as inorganic phosphate ions. The binding mode of the phosphate ion found in the BaLS structure is very similar to the phosphate-binding modes described in published LS structures. Each phosphate ion establishes electrostatic contacts with side-chain N atoms of Arg126 and hydrogen bonds with main-chain O and N and side-chain O atoms of Thr85, as well as with the main-chain N atom of Ala84 (Fig. 3 ▶ *f*). Both Arg126 and Thr85 are strictly conserved among all known LSs. It has been shown previously that mutation of the Arg126 residue to histidine or lysine reduced the enzymatic activity to 62% and 9%, respectively, while mutation to an uncharged residue resulted in inactive enzyme (Fischer *et al.*, 2003 ▶). In icosahedral LSs any two active sites are connected by twofold icosahedral axes and the distance between two *C*_2_ symmetry-related phosphate ions is 15.4 Å. These twofold-symmetric regions of the icosahedral assembly form pore-like structures which could serve as alternative exit paths for the product of the catalytic reaction (Ladenstein *et al.*, 2009 ▶).

### 3.4. Bisubstrate-type LS inhibitors

**Figure 4:**
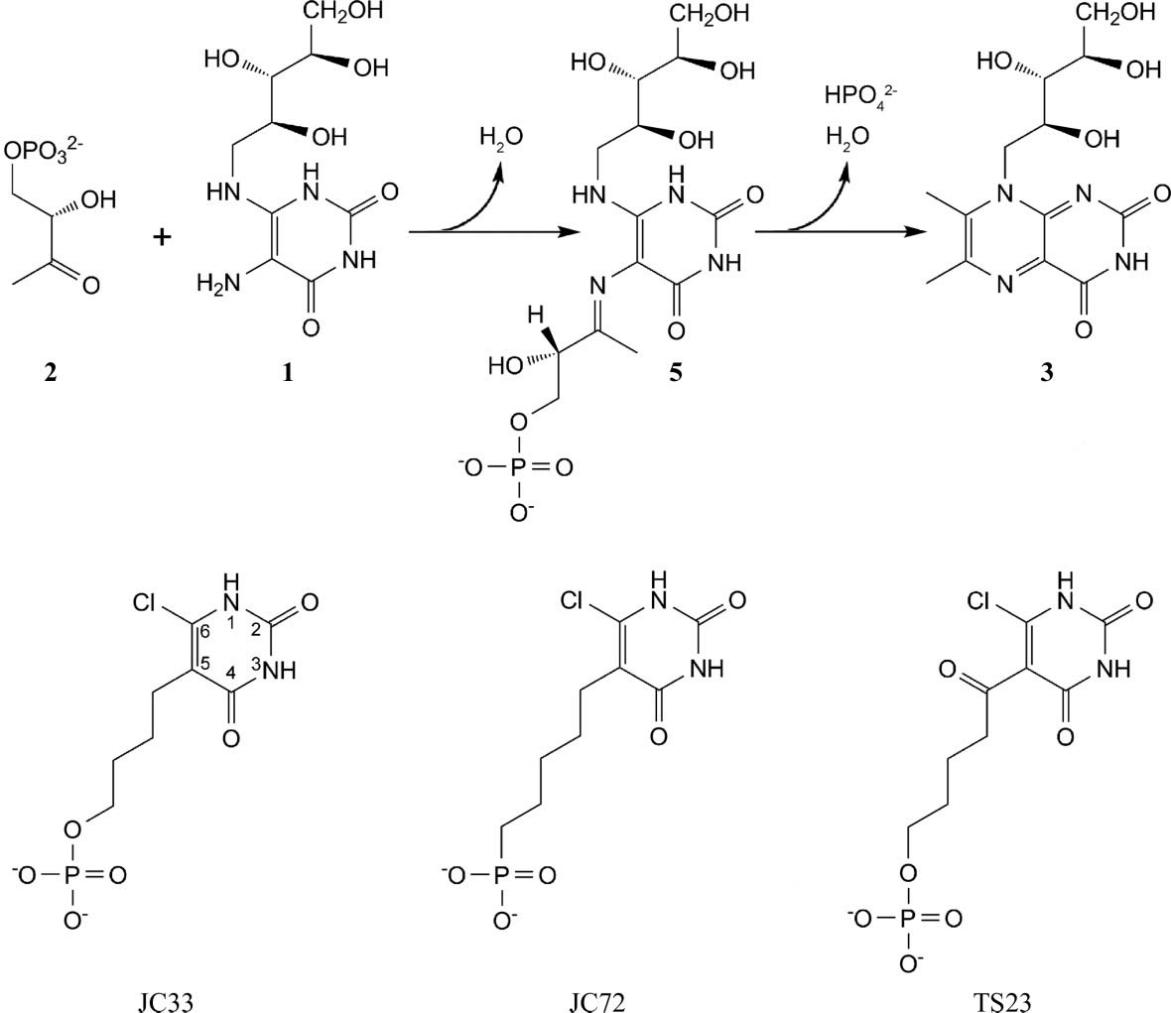
Top, reaction mechanism of lumazine synthase. Bottom, the inhibitors used in this study.

The complex reaction mechanism of LS has been studied in considerable detail. A crucial step is the initial formation of a covalent bond between the two substrate molecules, compounds **1** and **2** (Fig. 1 ▶), which results in the formation of the Schiff-base-type intermediate compound **5** (Fig. 4 ▶). This has raised the possibility of bisubstrate-type inhibitors or intermediate-analogue inhibitors which emulate the structural features of both substrates (*i.e.* of com­pounds **1** and **2**). Using 20 compounds of this structural type, we performed preliminary inhibition tests with BaLS. The three compounds with the highest inhibitory potential (compounds TS23, JC33 and JC72) were selected for detailed kinetic analysis.

### 3.5. Kinetic assay of lumazine synthase inhibitors

**Figure 5:**
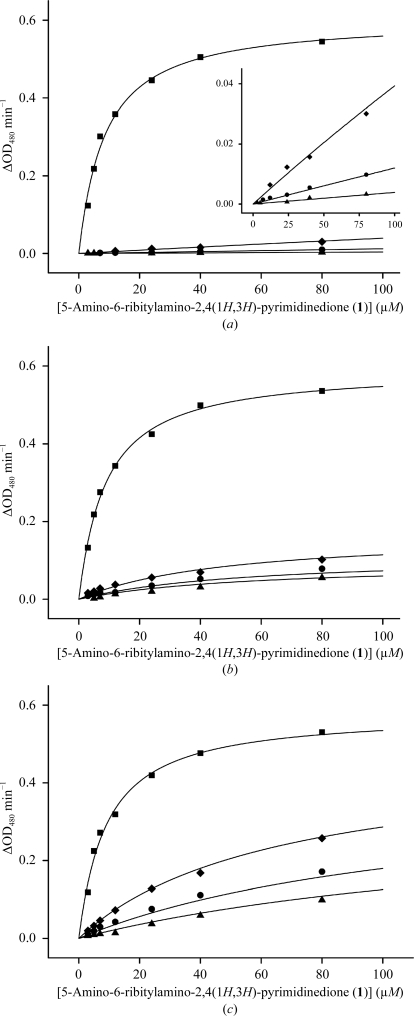
Kinetics plots for the inhibition of *B. anthracis* LS by the following inhibitors: JC33 (*a*), TS23 (*b*) and JC72 (*c*). The inhibitor concentrations were 0 µ*M* (squares), 5 µ*M* (diamonds), 15 µ*M* (circles) or 45 µ*M* (triangles). The inset in (*a*) shows an extended view of the same plot in the region ΔOD_408_ = 0 for inhibitor concentrations of 5, 15 and 45 µ*M*.

The LS inhibitors JC33, JC72 and TS23 were tested as inhibitors of recombinant BaLS using a kinetic assay (Fig. 5 ▶). The inhibition constants and inhibition mechanisms for these inhibitors are listed in Table 3 ▶.

The data show that all three compounds are potent inhibitors of the enzyme, with *K*_i_ values in the low nanomolar range. The most potent inhibitor is compound JC33 (*K*_i_ = 23 n*M*), which contains a phosphobutyl moiety as a side chain. The insertion of a carbonyl group into this side chain leads to compound TS23 with an oxopentylphosphate side chain and increases the *K*_i_ value sevenfold (to 140 n*M*) in comparison to JC33. A pentylphosphonate moiety in compound JC72 increases the *K*_i_ value by approximately 20-fold (to 430 n*M*) in comparison to JC33.

Previously, we reported that the alkylphosphate moiety plays an important role in the binding of certain inhibitors to the active site of MtLS (Cushman *et al.*, 2004 ▶; Morgunova *et al.*, 2005 ▶). The ribityl side chain of the tested inhibitors seemed to be equally important, at least with respect to inhibitors binding to MtLS. The fact that inhibitor JC33, which is devoid of a ribityl side chain, has a high inhibition potency for BaLS, with a *K*_i_ of 23 n*M*, makes it an attractive lead compound for the discovery of new anti-infective agents against anthrax.

### 3.6. Isothermal titration calorimetry

**Figure 6:**
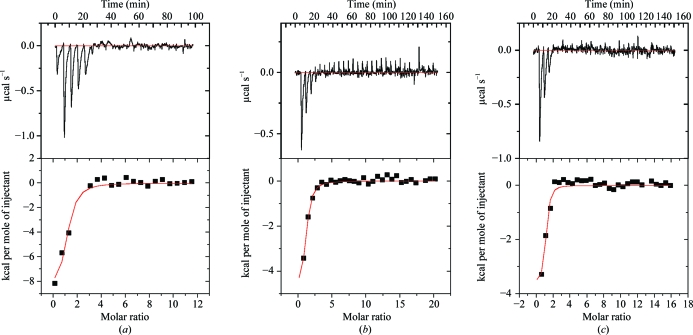
Raw calorimetric data (top) and derived binding isotherms (bottom) for the titration of lumazine synthase from *B. anthracis* with JC33 (*a*), JC72 (*b*) and TS23 (*c*). The filled squares in the binding isotherms represent the experimental values of the heat changes at each injection; the continuous lines represent the results of the data fitting to the chosen binding model. 1 cal = 4.186 J.

The thermodynamics of inhibitor binding by BaLS were studied by isothermal titration calorimetry (ITC). The apparent association constant, stoichiometry and binding enthalpy were derived from fitting of the binding isotherms. Fig. 6 ▶ shows typical calorimetric titration curves of BaLS in 50 m*M* phosphate buffer at pH 7.0 and *T* = 303 K. Thermodynamic characteristics such as the entropy and free energy of binding were deduced from the Gibbs–Helmholtz equation [Δ*G* = −*RT*ln(*K*_a_) = Δ*H* − *T*Δ*S*; see also §[Sec sec2]2]. All three binding reactions were found to be exothermic, with favourable negative changes of the binding enthalpy Δ*H*. Interestingly, all three binding reactions are characterized by positive changes in *T*Δ*S*. Thus, the small changes in enthalpy were compensated by corresponding favourable positive changes in entropy. The resulting free-energy values are similar for all three complexes. For comparison, the thermodynamic characteristics of complexes of JC33 with MtLS and *C. albicans* LS (CaLS) are presented in the two right-hand columns of Table 4 ▶. The dissociation constants of both complexes with pentameric enzymes are almost tenfold lower than for the icosahedral BaLS. The changes in free binding energy are higher in the com­plexes of pentameric enzymes compared with the BaLS–JC33 complex. The entropy changes for the binding of all three inhibitors by BaLS as well as the entropy change of the binding of JC33 by CaLS were found to be positive, whereas MtLS binds JC33 with an unfavourable negative change in entropy. The overlaid structures of the active sites of BaLS with CaLS and MtLS and sequence alignment showed that the position of the residue located near Arg126 in the active site is occupied by Gly132 in BaLS and by His148 in CaLS; however, this position is occupied by a negatively charged glutamate residue in MtLS. The binding of the inhibitors is accompanied by a dislocation of water molecules present in the active site of the unliganded enzyme. Thus, it seems unrealistic to attempt an interpretation of the positive entropy changes in molecular terms with respect to specific features of the inhibitors.

### 3.7. Structure-based modelling of the inhibitors in the active site of BaLS

**Figure 7:**
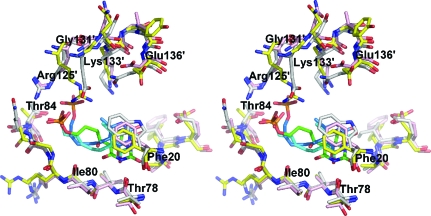
Stereo diagram of the active site of lumazine synthase from *B. anthracis* with docked models of JC33 (shown in cyan), JC72 (shown in magenta) and TS23 (shown in orange); residues of the adjacent symmetry-equivalent subunit are labelled with primes.

No major conformational changes have been found to accompany the binding of inhibitors to the known LS orthologues, with the exception of the aromatic amino-acid residue (Phe or Tyr) in position 21 (Ladenstein *et al.*, 1988 ▶; Meining *et al.*, 2000 ▶; Gerhardt *et al.*, 2002 ▶; Zhang *et al.*, 2003 ▶; Klinke *et al.*, 2005 ▶; Morgunova *et al.*, 2005 ▶, 2006 ▶). The respective benzenoid ring is oriented parallel to the plane of the aromatic group of the inhibitor, whereas the same group in the apo structures assumed different conformations in different subunits of the same molecule (Persson *et al.*, 1999 ▶; Braden *et al.*, 2000 ▶; Zhang *et al.*, 2001 ▶; Morgunova *et al.*, 2007 ▶; present structure). By using the structural information from a substantial number of LS structures with phosph(on)oalkyl ligands, we have initiated docking studies of inhibitors in the active site of BaLS and we present here the hypothetical binding modes for complexes of BaLS with the three inhibitors described above. The docking calculations revealed different possible positions and orientations of binding of all three inhibitors (Fig. 7 ▶). The active site of LSs is rather large and can be occupied by inhibitors which are more bulky than the derivatives of the sub­strate, com­pound **1** (Fig. 1 ▶), presented in this work (for example, lumazine or purine derivatives containing both ribityl and aliphatic side chains). The hypothetical binding modes of all three inhibitors are very similar to that found in the structure of the MtLS–JC33 complex with respect to the different amino acids in the active site. The aromatic rings of the inhibitors are found in close proximity to the benzene ring of Phe21 at a distance of 4.3–4.6 Å and the phosphate/phosphonate moieties are exposed to the locations of the side chains of Arg126 and Thr85. Slightly different conformations of the inhibitors were observed with different lengths of their aliphatic chains, which can be explained by the packing strain exerted on those chains by the available space. Molecular-dynamics calculations followed by energy minimization of the favourable models performed with *CNS* moved the side chain of Phe21 into a conformation that is parallel to the position of the aromatic ring of the inhibitors, as expected. Thus, the pyrimidine rings of all three inhibitors form stacking aromatic interactions with the benzene ring of the phenylalanine residue, although the rings of the inhibitors are slightly shifted with respect to each other. The phosphonate moiety of JC72 occupies the same position as the phosphate moiety of JC33, although the pyrimidine ring of JC72 is slightly shifted relative to the pyrimidine ring of JC33. A possible explanation is the presence of an additional C atom in the aliphatic chain of JC72 in com­parison with JC33. The compound TS23 has the same-length aliphatic chain as JC72 with an additional carbonyl O atom attached to the aliphatic chain in the position next to the pyrimidine ring. The binding mode of this inhibitor shows a different packing of the aliphatic chain in the active-site space, with the phosphate moiety shifted slightly closer to the side chain of Arg126. This phosphate moiety is involved in ion-pair bonding with the guanidyl group of the side chain of Arg126 and in addition interacts with the side chain of Thr85 (Fig. 7 ▶). Thus, the differences found in the thermodynamic constants of binding of the JC72 and TS23 inhibitors compared with JC33, as well as the lower binding constant, can be explained by increasing difficulty in packing the longer aliphatic chain of those inhibitors into the active-site space. This conclusion agrees with our previous results of the investigation of the optimum length of the aliphatic chain of purinetrione inhibitors, which showed that the most favourable binding characteristics are shown by the compounds with an aliphatic chain consisting of 4–5 C atoms (Morgunova *et al.*, 2006 ▶, 2007 ▶).

## Supplementary Material

PDB reference: lumazine synthase, 1vsx

PDB reference: 1vsw

PDB reference: 3jv8

## Figures and Tables

**Table 1:** The oligonucleotides used for the cloning of lumazine synthase from *B. anthracis*

Primer	Novel restriction site	Sequence (5′→3′)
BARibH-Rbs-*Eco*RI	*Eco*RI	ATA ATA GAA TTC ATT AAA GAG GAG AAA TTA ACT
BARibH-*Bam*HI-Hi	*Bam*HI	TAT TAT GGA TCC TTA TGC CCA ATG TTT TGA TAA

**Table 2:** Data-collection and refinement statistics Values in parentheses are for the outer shell.

Data collection	
Resolution limits (Å)	40–3.5 (3.69–3.5)
No. of observed reflections	1028468 (150011)
No. of unique reflections	196243 (28170)
Completeness (%)	93.3 (93.5)
Multiplicity	5.3 (5.3)
〈*I*/σ(*I*)〉	5.2 (2.2)
*R*_p.i.m._† (%)	12.9 (37.4)
Refinement	
Resolution range (Å)	15–3.5
Non-H protein atoms	101742
Non-H ion atoms	450
*R*_cryst_ overall‡ (%)	23.7
*R*_free_§ (%)	28.4
Ramachandran plot	
Most favourable regions (%)	85.3
Allowed regions (%)	14.7
Generously allowed regions (%)	0.0
R.m.s. standard deviation	
Bond lengths (Å)	0.002
Bond angles (°)	0.54

† *R*_p.i.m._ is the precision-indicating (multiplicity-weighted) *R*_merge_ (Diederichs & Karplus, 1997 ▶).

‡ *R*_cryst_ = 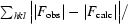 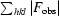.

§ *R*_free_ is the cross-validation *R* factor computed for a test set of 5% of the unique reflections.

**Table 3:** Inhibition constants for *B. anthracis* lumazine synthase Experiments were conducted with recombinant BaLS. The assays were performed with the concentration of compound **2** (see Fig. 1 ▶) held constant, while the concentration of compound **1** was varied. Reaction mixtures contained 100 m*M* Tris–HCl pH 7.0, 100 m*M* NaCl and 5 m*M* DTT. For the competitive mechanism it is assumed that the inhibitor binds at the substrate (compound **1**) binding site and inactivates the enzyme completely. This model is described by the following chemical equations: E + S ↔ ES (substrate binding at enzyme), *K*_s_ is the dissociation constant for this equilibrium; ES → E + P (conversion of the enzyme–substrate complex into enzyme and product), *k*_cat_ is the rate constant for this process; E + I ↔ EI (binding of inhibitor at the binding site for the substrate, *i.e.* compound **1**), *K*_i_ is the inhibitor-dissociation constant for this equilibrium. For the partial mechanism it is assumed that the inhibitor binds at the binding site for compound **1** and inactivates the enzyme completely. In parallel it can bind at the enzyme away from the binding site for compound **1**. In this case it can only partially inactivate the enzyme. This model is described by the following chemical equations: E + S ↔ ES (substrate binding by enzyme), *K*_s_ is the dissociation constant for this equilibrium; ES → E + P (conversion of the enzyme–substrate complex into enzyme and product), *k*_cat_ is the rate constant for this process; E + I ↔ EI (binding of inhibitor at the binding site for compound **1**), *K*_i_ is the inhibitor-dissociation constant for this equilibrium; ES + I ↔ ESI (binding of the inhibitor away from the binding site for compound **1**), *K*_is_ is the inhibitor-dissociation constant for this equilibrium; ESI → E + I + P [conversion of the triple complex (enzyme–substrate–inhibitor) into enzyme, inhibitor and product], *k*′_cat_ is the rate constant for this process. A numerical solution to the problem of minimizing a least-squares function over a space of reaction parameters was found using the Levenberg–Marquardt algorithm.

Compound	Mechanism	*K*_s_ (µ*M*)	*k*_cat_ (min^−1^)	*K*_i_ (µ*M*)	*K*_is_ (µ*M*)	*k*′_cat_ (min^−1^)
JC33	Competitive	8.0 ± 0.5	0.60 ± 0.01	0.023 ± 0.006		
JC72	Partial	8.5 ± 0.5	0.60 ± 0.01	0.43 ± 0.06	9.5 ± 3.5	0.28 ± 0.09
TS23	Partial	8.3 ± 0.4	0.60 ± 0.01	0.14 ± 0.03	0.85 ± 0.2	0.08 ± 0.01

**Table 4:** Thermodynamic parameters of binding of different inhibitors to *B. anthracis* lumazine synthase 1 kcal = 4.186 kJ.

	BaLS–TS23	BaLS–JC72	BaLS–JC33	MtLS–JC33 (Morgunova *et al.*, 2006 ▶)	CaLS–JC33 (Morgunova *et al.*, 2007 ▶)
No. of sites *n*	0.97 ± 0.06	0.98 ± 0.12	0.98 ± 0.04	0.98 ± 0.04	1.15 ± 0.03
Dissociation constant *K*_d_ (µ*M*)	2.02 ± 0.03	3.94 ± 0.01	1.75 ± 0.04	0.72 ± 0.02	0.15 ± 0.04
Binding enthalpy Δ*H* (kcal mol^−1^)	−3.70 ± 0.27	−5.29 ± 0.8	−7.50 ± 0.07	−10.52 ± 0.11	−6.98 ± 0.24
*T*Δ*S*† (kcal mol^−1^)	4.19 ± 0.27	2.20 ± 0.79	0.47 ± 0.07	−2.00 ± 0.12	2.44 ± 0.25
Free energy of binding *ΔG*† (kcal mol^−1^)	−7.89 ± 0.01	−7.49 ± 0.01	−7.98 ± 0.02	−8.52 ± 0.05	−9.38 ± 0.01

† The entropy of the binding reactions (Δ*S*) and the free-energy change (Δ*G*) are obtained from the relation Δ*G* = −*RT*ln(*K*_a_) = Δ*H* − *T*Δ*S*; the estimated errors of *T*Δ*S* and Δ*G* are obtained from the relations σ_Δ*G*_ = 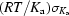 and *T*σ_Δ*S*_ = {[(*d*Δ*S*/*d*Δ*G*)σ_Δ*G*_]^2^ + [(*d*Δ*S*/*d*Δ*H*)σ_Δ*H*_]^2^}^1/2^ = [(σ_Δ*G*_)^2^ + (σ_Δ*H*_)^2^]^1/2^, respectively (Taylor, 1997 ▶).

## References

[bb1] Adams, P. D., Grosse-Kunstleve, R. W., Hung, L.-W., Ioerger, T. R., McCoy, A. J., Moriarty, N. W., Read, R. J., Sacchettini, J. C., Sauter, N. K. & Terwilliger, T. C. (2002). *Acta Cryst.* D**58**, 1948–1954. 10.1107/s0907444902016657 12393927

[bb2] Bourenkov, G. P. & Popov, A. N. (2006). *Acta Cryst.* D**62**, 58–64. 10.1107/S0907444905033998 16369094

[bb3] Braden, B. C., Velikovsky, C. A., Cauerhff, A. A., Polikarpov, I. & Goldbaum, F. A. (2000). *J. Mol. Biol.* **297**, 1031–1036. 10.1006/jmbi.2000.3640 10764570

[bb4] Brünger, A. T., Adams, P. D., Clore, G. M., DeLano, W. L., Gros, P., Grosse-Kunstleve, R. W., Jiang, J.-S., Kuszewski, J., Nilges, M., Pannu, N. S., Read, R. J., Rice, L. M., Simonson, T. & Warren, G. L. (1998). *Acta Cryst.* D**54**, 905–921. 10.1107/s0907444998003254 9757107

[bb5] Chen, J., Illarionov, B., Bacher, A., Fischer, M., Haase, I., Georg, G., Ye, Q. Z., Ma, Z. & Cushman, M. (2005). *Anal. Biochem.* **338**, 124–130. 10.1016/j.ab.2004.11.033 15707942

[bb6] Collaborative Computational Project, Number 4 (1994). *Acta Cryst.* D**50**, 760–763.

[bb7] Cushman, M., Jin, G., Sambaiah, T., Illarionov, B., Fischer, M., Ladenstein, R. & Bacher, A. (2005). *J. Org. Chem.* **70**, 8162–8170. 10.1021/jo051332v PMC2548293 16277343

[bb8] Cushman, M., Mavandadi, F., Kugelbrey, K. & Bacher, A. (1997). *J. Org. Chem.* **62**, 8944–8947.

[bb9] Cushman, M., Mavandadi, F., Yang, D., Kugelbrey, K., Kis, K. & Bacher, A. (1999). *J. Org. Chem.* **64**, 4635–4642. 10.1021/jo9821731 11674533

[bb10] Cushman, M., Mihalic, J. T., Kis, K. & Bacher, A. (1999*a*). *Bioorg. Med. Chem. Lett.* **9**, 39–42. 10.1016/s0960-894x(98)00687-8 9990453

[bb11] Cushman, M., Mihalic, J. T., Kis, K. & Bacher, K. (1999*b*). *J. Org. Chem.* **64**, 3838–3845.

[bb12] Cushman, M., Sambaiah, T., Jin, G., Illarionov, B., Fischer, M. & Bacher, A. (2004). *J. Org. Chem.* **69**, 601–612. 10.1021/jo030278k 14750781

[bb13] Cushman, M., Yang, D., Gerhardt, S., Huber, R., Fischer, M., Kis, K. & Bacher, A. (2002). *J. Org. Chem.* **67**, 5807–5816. 10.1021/jo0201631 12153285

[bb14] Cushman, M., Yang, D., Kis, K. & Bacher, A. (2001). *J. Org. Chem.* **66**, 8320–8327. 10.1021/jo010706r 11735509

[bb15] DeLano, W. L. (2002). *The PyMOL Molecular Viewer.* http://www.pymol.org.

[bb16] Diederichs, K. & Karplus, A. P. (1997). *Nature Struct. Biol.* **4**, 269–275. 10.1038/nsb0497-269 9095194

[bb17] Emsley, P. & Cowtan, K. (2004). *Acta Cryst.* D**60**, 2126–2132. 10.1107/S0907444904019158 15572765

[bb18] Fischer, M., Haase, I., Kis, K., Meining, W., Ladenstein, R., Cushman, M., Schramek, N., Huber, R. & Bacher, A. (2003). *J. Mol. Biol.* **326**, 783–793. 10.1016/s0022-2836(02)01473-0 12581640

[bb19] Gerhardt, S., Haase, I., Steinbacher, S., Kaiser, J. T., Cushman, M., Bacher, A., Huber, R. & Fischer, M. (2002). *J. Mol. Biol.* **318**, 1317–1329. 10.1016/s0022-2836(02)00116-x 12083520

[bb20] Jones, T. A., Zou, J.-Y., Cowan, S. W. & Kjeldgaard, M. (1991). *Acta Cryst.* A**47**, 110–119. 10.1107/s0108767390010224 2025413

[bb21] Kabsch, W. (1988). *J. Appl. Cryst.* **21**, 916–924.

[bb22] Kabsch, W. (2010). *Acta Cryst.* D**66**, 125–132. 10.1107/S0907444909047337 PMC2815665 20124692

[bb23] Kis, K. & Bacher, A. (1995). *J. Biol. Chem.* **270**, 16788–16795. 10.1074/jbc.270.28.16788 7622491

[bb24] Kis, K., Volk, R. & Bacher, A. (1995). *Biochemistry*, **34**, 2883–2892. 10.1021/bi00009a019 7893702

[bb25] Klinke, S., Zylberman, V., Vega, D. R., Guimaraes, B. G., Braden, B. C. & Goldbaum, F. A. (2005). *J. Mol. Biol.* **353**, 124–137. 10.1016/j.jmb.2005.08.017 16165152

[bb26] Kuzmic, P. (1996). *Anal. Biochem.* **237**, 260–273. 10.1006/abio.1996.0238 8660575

[bb27] Ladenstein, R., Meining, W., Zhang, X., Fischer, M. & Bacher, A. (2009). *Biotechnol. Biotechnol. Equip.* **23**, 1153–1161.

[bb28] Ladenstein, R., Schneider, M., Huber, R., Bartunik, H. D., Wilson, K., Schott, K. & Bacher, A. (1988). *J. Mol. Biol.* **203**, 1045–1070. 10.1016/0022-2836(88)90128-3 3145341

[bb29] Liao, D. I., Calabrese, J. C., Wawrzak, Z., Viitanen, P. V. & Jordan, D. B. (2001). *Structure*, **9**, 11–18. 10.1016/s0969-2126(00)00550-5 11342130

[bb30] Meining, W., Mörtl, S., Fischer, M., Cushman, M., Bacher, A. & Ladenstein, R. (2000). *J. Mol. Biol.* **299**, 181–197. 10.1006/jmbi.2000.3742 10860731

[bb31] Morgunova, E., Illarionov, B., Sambaiah, T., Haase, I., Bacher, A., Cushman, M., Fischer, M. & Ladenstein, R. (2006). *FEBS J.* **273**, 4790–4804. 10.1111/j.1742-4658.2006.05481.x 16984393

[bb32] Morgunova, E., Meining, W., Cushman, M., Illarionov, B., Haase, I., Jin, G., Bacher, A., Cushman, M., Fischer, M. & Ladenstein, R. (2005). *Biochemistry*, **44**, 2746–2758. 10.1021/bi047848a 15723519

[bb33] Morgunova, E., Saller, S., Haase, I., Cushman, M., Bacher, A., Fischer, M. & Ladenstein, R. (2007). *J. Biol. Chem.* **282**, 17231–17241. 10.1074/jbc.M701724200 17446177

[bb34] Morris, G. M., Goodsell, D. S., Halliday, R. S., Huey, R., Hart, W. E., Belew, R. K. & Olson, A. J. (1998). *J. Comput. Chem.* **19**, 1639–1662.

[bb35] Persson, K., Schneider, G., Jordan, D. B., Viitanen, P. V. & Sandalova, T. (1999). *Protein Sci.* **8**, 2355–2365. 10.1110/ps.8.11.2355 PMC2144189 10595538

[bb36] Pettersen, E. F., Goddard, T. D., Huang, C. C., Couch, G. S., Greenblatt, D. M., Meng, E. C. & Ferrin, T. E. (2004). *J. Comput. Chem.* **25**, 1605–1612. 10.1002/jcc.20084 15264254

[bb37] Ritsert, K., Huber, R., Turk, D., Ladenstein, R., Schmidt-Bäse, K. & Bacher, A. (1995). *J. Mol. Biol.* **253**, 151–167. 10.1006/jmbi.1995.0542 7473709

[bb38] Stüber, D., Matile, H. & Garotta, G. (1990). *Immunological Methods*, Vol. IV, edited by I. Lefkovits & B. Pernis, pp. 121–152. New York: Academic Press.

[bb39] Taylor, J. R. (1997). *An Introduction to Error Analysis: The Study of Uncertainties in Physical Measurements*, 2nd ed. Sausalito: University Science Books.

[bb41] Wang, J., Wang, W., Kollman, P. A. & Case, D. A. (2006). *J. Mol. Graph. Model.* **25**, 247–260. 10.1016/j.jmgm.2005.12.005 16458552

[bb43] Zhang, X., Meining, W., Cushman, M., Haase, I., Fischer, M., Bacher, A. & Ladenstein, R. (2003). *J. Mol. Biol.* **328**, 167–182. 10.1016/s0022-2836(03)00186-4 12684006

[bb44] Zhang, X., Meining, W., Fischer, M., Bacher, A. & Ladenstein, R. (2001). *J. Mol. Biol.* **306**, 1099–1114. 10.1006/jmbi.2000.4435 11237620

[bb45] Zhang, Y., Illarionov, B., Morgunova, E., Jin, G., Bacher, A., Fischer, M., Ladenstein, R. & Cushman, M. (2008). *J. Org. Chem.* **73**, 2715–2724. 10.1021/jo702631a 18331058

